# Supplementary material for: A new machine learning method for rainfall classification: temporal random tree
Source: PeerJ Comput Sci. 2025 Jul 14;11:e3022. doi: 10.7717/peerj-cs.3022 (PMC12453857; doi:10.7717/peerj-cs.3022)
Supplement: Supplemental Information 3 [file peerj-cs-11-3022-s003.docx]

**Table A1.** Abbreviations

| **Abbreviation** | **Description** |
| --- | --- |
| ANN | Artificial neural network |
| ARIMA | Autoregressive integrated moving average model |
| BLSTM | Bidirectional long short-term memory |
| CC | Correlation coefficient |
| CNN | Convolutional neural network |
| COE | Coefficient of efficiency |
| CORR | Correlation coefficient |
| CSI | Critical success index |
| DBM | Deep belief network |
| DFFNet | Dual-branch feature fusion model |
| DNN | Deep neural networks |
| DWT | Discrete wavelet transform |
| FAR | False alarm rate |
| GIS | Geographic information system |
| GPM | Global precipitation measurement |
| GRU | Gated recurrent unit |
| GWR | Geographically weighted regression |
| HSS | Heidke skill score |
| IA | Index of agreement |
| IMERG | Integrated multi satellite retrievals |
| KGE | Kling–Gupta efficiency |
| KNN | K-nearest neighbors |
| LI | Linear interpolation |
| LMI | Legates–McCabe index |
| LR | Linear regression |
| LSTM | Long short-term memory |
| MAE | Mean absolute error |
| MAPE | Mean absolute percentage error |
| MAPLE | McGill algorithm for precipitation nowcasting by Lagrangian extrapolation |
| MARS | Multivariate adaptive regression splines |
| MBE | Mean bias error |
| ML | Machine learning |
| MLP | Multilayer perceptron |
| MLR | Multiple linear regression |
| NB | Naive Bayes |
| NE | Normalized error |
| NPV | Negative predictive value |
| NSE | Nash-Sutcliffe efficiency |
| NSEC | Nash-Sutcliffe efficiency coefficient |
| OI | Overall index |
| PCA | Principal component analysis |
| PCC | Pearson correlation coefficient |
| PEMR | Percent error in maximum rainfall |
| POD | Probability of detection |
| PPMCC | Pearson product-moment correlation coefficients |
| PSO | Particle swarm optimization |
| RAE | Relative absolute error |
| RBF | Relative forecast bias |
| RF | Random forest |
| RFB | Relative forecast bias |
| RIAA | Agroclimatic Information network of Andalusia |
| RIPPER | Repeated incremental pruning to produce error reduction |
| rMAE | Relative MAE |
| RMSE | Residual mean square error |
| RNN | Recurrent neural network |
| rRMSE | Relative RMSE |
| RSR | RMSE-observations standard deviation ratio |
| RT | Random tree |
| RVMs | Relevant vector machines |
| SIG | Significance value |
| SSIM | Structural similarity index measure |
| SVC | Support vector classification |
| SVM | Support vector machine |
| SVR | Support vector regression |
| TabNet | Tabular learning neural network |
| TDR | True detection rate |
| TRT | Temporal random tree |
| TS | Threat score |
| WPD-ELM | Wavelet packet decomposition-extreme learning machine |
| WRIS | Water resources information system |
| XGBoost | Extreme gradient boosting |
